# Automated Contouring and Planning in Radiation Therapy: What Is ‘Clinically Acceptable’?

**DOI:** 10.3390/diagnostics13040667

**Published:** 2023-02-10

**Authors:** Hana Baroudi, Kristy K. Brock, Wenhua Cao, Xinru Chen, Caroline Chung, Laurence E. Court, Mohammad D. El Basha, Maguy Farhat, Skylar Gay, Mary P. Gronberg, Aashish Chandra Gupta, Soleil Hernandez, Kai Huang, David A. Jaffray, Rebecca Lim, Barbara Marquez, Kelly Nealon, Tucker J. Netherton, Callistus M. Nguyen, Brandon Reber, Dong Joo Rhee, Ramon M. Salazar, Mihir D. Shanker, Carlos Sjogreen, McKell Woodland, Jinzhong Yang, Cenji Yu, Yao Zhao

**Affiliations:** 1 Department of Radiation Physics, University of Texas MD Anderson Cancer Center, Houston, TX 77030, USA; 2 The University of Texas MD Anderson Cancer Center UTHealth Houston Graduate School of Biomedical Sciences, Houston, TX 77030, USA; 3 Department of Imaging Physics, Department of Radiation Physics, University of Texas MD Anderson Cancer Center, Houston, TX 77030, USA; 4 Department of Radiation Oncology, University of Texas MD Anderson Cancer Center, Houston, TX 77030, USA; 5 The University of Queensland, Saint Lucia 4072, Australia; 6 The University of Texas MD Anderson Cancer Center, Houston, TX 77030, USA; 7 Department of Physics, University of Houston, Houston, TX 77004, USA; 8 Department of Computer Science, Rice University, Houston, TX 77005, USA

**Keywords:** radiotherapy treatment planning, artificial intelligence, quality assurance

## Abstract

Developers and users of artificial-intelligence-based tools for automatic contouring and treatment planning in radiotherapy are expected to assess clinical acceptability of these tools. However, what is ‘clinical acceptability’? Quantitative and qualitative approaches have been used to assess this ill-defined concept, all of which have advantages and disadvantages or limitations. The approach chosen may depend on the goal of the study as well as on available resources. In this paper, we discuss various aspects of ‘clinical acceptability’ and how they can move us toward a standard for defining clinical acceptability of new autocontouring and planning tools.

## 1. Introduction

Recent advances in artificial intelligence (AI) and other automation techniques have generated a wave of new tools for automated contouring and treatment planning in radiation therapy. This wave, which includes contributions from academic researchers, established vendors, and startup companies, offers many potential advantages, such as improved consistency, quality, and efficiency when compared with manual contouring or planning.

**Figure 1:**
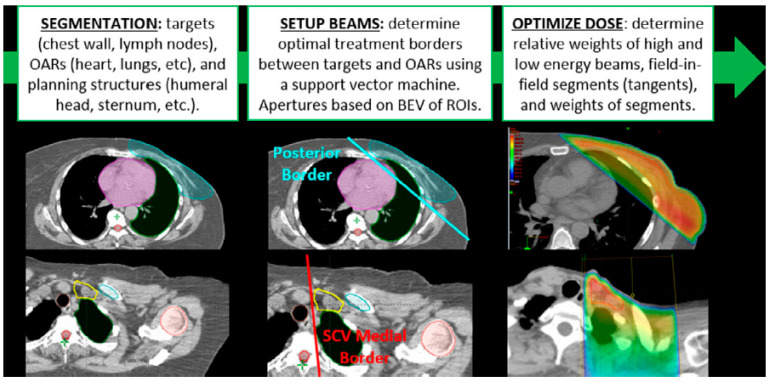
A typical automation workflow starts with automated segmentation of targets and organs at risk (OARs), followed by treatment planning (here described by beam setup and dose optimization). The individual tasks may be automated separately or a complete end-to-end process with no user intervention until the end. This example is from Kisling et al. [1].

Traditional radiotherapy workflow starts with contouring (segmentation) of a CT image set, followed by positioning and optimization of radiotherapy beams (i.e., treatment planning). Automation typically follows the same workflow, with each task automated separately. The two automation tasks of segmentation and planning may be combined into a single end-to-end workflow. An example of a combined workflow is shown in Figure 1 for automation of treatment planning for post-mastectomy breast cancer patients. Whether automating individual tasks or complete end-to-end systems, one challenge faced by both developers and consumers is assessment of value and utility in healthcare systems. How do we know when a new autocontouring or autoplanning tool is “clinically acceptable”? This concept remains vaguely defined and lacks standardized metrics for its assessment. The challenge lies in the fact that clinical acceptability is a multi-faceted concept that attempts to describe whether introduction of a new tool meets the needs of a clinical team in achieving a high level of clinical performance. Assessment of clinical acceptability relies on finding relevant intermediate metrics focused on three main pillars: (1) quantitative comparison with manually drawn clinical contours or treatment plans, (2) qualitative subjective reviews by subject matter experts (i.e., radiation oncologists), and (3) ultimate measures of efficiency and (predicted) impact on tumor control or similar clinically meaningful outcomes. Each one of these assessment approaches varies regarding resources necessary for completion and how directly it is linked to “clinical acceptability”.

The purpose of this paper is to provide a detailed review of the different assessment approaches and metrics that can be used to evaluate the clinical acceptability of autocontouring and autoplanning tools so that developers and consumers can better evaluate, deploy, and improve upon them. We start by following the workflow of Figure 1, followed by a discussion of other factors that can affect the clinical acceptability of a new tool.

## 2. Automated Segmentation

In current clinical practice, radiologists, dosimetrists, or physicists delineate anatomic structures manually before radiation treatment planning. Manual delineation is subject to interobserver variability (IOV), which includes manual steadiness, attentiveness, expertise, and interpretation of the observer. The main uncertainty in radiotherapy treatment planning for most tumor locations has been demonstrated to be the IOV in target volume contouring, which may lead to systematic errors in dose administration that could affect local disease control [2]. Moreover, intra- and inter-institution IOVs have been found to be considerable in manual segmentation of both organs at risk (OARs) and targets [3,4,5]. Apart from its use in formulating guidelines and training [6], autosegmentation has been shown to reduce IOV in volume delineation [7]. Manual modification of autosegmentation results leads to improved volume consistency in endometrial cancers [8,9]. Reported IOV of autosegmentations in consistency studies largely depends on expertise of involved observers, models, and treatment sites. In one study of cervical cancer, variations between automatically generated contours and manually generated contours from experienced radiation oncologists were smaller than variations between experienced-mid-career or experienced-early-career radiation oncologists [10]. In abdominal cancers, inferior contour consistency was reported between 3D U-Net models and experts [11]. Non-expert segmentations have also been suggested to be practical and cost-effective AI inputs; in one study, five non-experts could produce consensus segmentations for most regions of interest with performance close to that of experts [12]. As a result, autosegmentation models have been deployed in clinics to complement manual contouring processes.

In this section we describe different approaches to evaluating autocontouring algorithms: Quantitative, qualitative, dosimetric, effect on efficiency, and predicted effect on patient outcomes; 

### 2.1. Quantitative Evaluation of Autocontours

Several quantitative approaches are available to evaluate contour accuracy. Most of the contouring accuracy metrics in the published literature are either volumetric overlap metrics, such as Dice similarity coefficients (DSCs) [13,14] and Jaccard; distance metrics such as Hausdorff distance (HD) [15], which is commonly reported as either a maximum or the 95th percentile, or mean surface distance (MSD) [16]; or, more recently, surface agreement, such as surface DSC [17]. DSC and Jaccard are ratios of contour overlap to overall volume and range from 0 (no overlap) to 1 (perfect structure agreement). HD is determined by calculating the distance from each point on the boundary of one surface to the nearest point on the boundary of the other structure and then selecting either the single largest distance or the 95th percentile of the distribution of distances (to be less sensitive to outliers). Mean surface distance is calculated similarly but with the mean of all distances returned. Distance metrics have no upper limit, and a smaller value corresponds to better agreement. Finally, surface DSC is calculated similarly to volumetric DSC but with only the ratio of the overlapping surfaces to total surface areas.

Recent studies have indicated that surface metrics are better for estimating clinically acceptable contours than either volumetric or distance metrics. Rhee et al. evaluated 13 metrics (DSC, HD, mean SD, and surface DSC with 1 to 10 mm margins) in a cohort of 49 female pelvis cases to find the most accurate similarity metric and found that surface DSC with 1 to 3 mm margins outperformed the others with respect to clinical acceptability [18]. A similar study of 20 lung cancer patients found that surface DSC and another surface metric they introduced, called added path length, were better able to demonstrate clinical contouring time savings, applicability, and quality of automatically generated contours compared with DSC or HD [19].

### 2.2. Qualitative Evaluation of Autocontours

**Figure 2:**
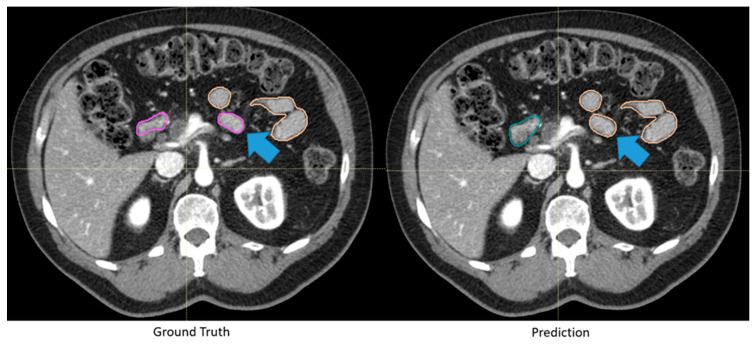
Automatically generated duodenum and small bowel contours with low Dice similarity coefficient (0.61) but were deemed clinically acceptable. Although the automatically generated contour (green: duodenum, yellow: small bowel) deviated from the “ground truth” (pink: duodenum, yellow: small bowel), as highlighted by the blue arrows, the difference was deemed insignificant by clinicians because of the identical dose constraints between the two segmented organs.

Because clinicians are often the end users of autosegmentation models, quantitative evaluations may not capture the full picture of clinical utility of models. Results of quantitative evaluation of generated contours may not indicate their clinical acceptability (Figure 2). Therefore, a common practice in related literature is to assess one or more clinicians’ perspectives of the automatically generated contours. This assessment is most commonly completed with Likert scales and Turing tests. Likert scales measure clinical usability of contours by ranking perceptual quality of contours on a predetermined scale; Turing tests evaluate the ability of the clinician to distinguish between manually and automatically created contours. The benefit of using these qualitative metrics is that they are more closely associated with clinical usability of contours. The main limitation of these metrics is that they are subjective and prone to human bias. Moreover, clinicians are accustomed to having access to additional information, such as physical exam, endoscopic data, and other images, that are often not available in these studies. These considerations can also affect quantitative assessments when comparing autocontours with manual contours (which were created including consideration of these additional data).

Although Likert scales always rate clinical usability of contours on a scale, the range of this scale varies. For example, Cha et al. used a 3-point Likert scale denoting that segmentation was either acceptable, unacceptable with minor edits required, or unacceptable with major edits required [20]. Zhong et al. used a 4-point scale in which the “acceptable” category was split into acceptable with precise contours and acceptable with small edits desired [21]. Wong et al. used two 5-point scales: one rated the number of edits required for clinical acceptability and the other the clinician’s satisfaction with the contour [22]. With such a wide variety of scales to choose from, we recommend a 5-point scale that was used in our clinical deployment [23] (Table 1). Other clinical evaluations rely on Turing test concepts, asking clinicians to distinguish between manually and automatically segmented structures [24]. Anderson et al. asked clinicians to indicate preferences between automatic and manually generated contours on a blinded basis [25]. Hoang Duc et al. [26] used a combination of Likert and Turing tests that asked physicians to rate manual and automatic segmentations on a blinded basis to validate the system’s performance.

### 2.3. Dosimetric Evaluation of Autocontours

Although quantitative metrics and qualitative evaluations such as those described above are standardized performance indicators of autosegmentation accuracy, only a few studies have robustly quantified contour uncertainty that translated into more clinical metrics, such as dose coverage. The standard approach to assess dose difference is to use constraints based on NRG/Radiation Therapy Oncology Group (RTOG) guidelines [27]. A difference of <1 Gy in dose and 1% in the volume of an OAR would also be deemed clinically acceptable [28].

The dosimetric impact of autosegmentation has been characterized as small and clinically acceptable in a few studies. Rigaud et. al. compared dose–volume histogram (DVH) parameters from plans for patients with cervical cancer optimized by automated or manual contouring. That group found that autosegmentations led to lower planning dose, but the difference was within 1% and 1 Gy, and weak-to-moderate correlations were found between DSC and clinical metrics such as dose to 98% of target volume (D98%) and target volume treated to 42.75 Gy or more (V42.75Gy) [29]. Similarly, Dong et al. assessed dosimetric impact of autocontours based on a general adversarial network (GAN) design in 20 plans for patients receiving stereotactic body radiation therapy (SBRT) to the lung; these investigators found the mean dose difference to be within 0.155 Gy for five OARs [30]. No statistical differences in dose metrics were found when the doses from automated contours of bladder and rectum in patients with prostate cancer were compared with their manual contour counterparts [31,32]. Vaassen et al. also found dose differences in OARs of patients with lung cancer from automatically generated contours that were either identical to or smaller than intraobserver variability [33].

In contrast, some studies found that autosegmentation significantly affected dose metrics. Thor et al. studied the impact of contouring inconsistencies in a clinical trial (heart doses in RTOG 0617) by comparing deep-learning-segmented hearts and trial hearts [34]. That group found that deep-learning-based contours resulted in higher doses to heart, as quantified by a difference of 3 Gy in mean heart dose, 5% in V30%, and 2% in V5% [34]. Mao et al. investigated the effects of an adaptive radiation therapy (ART) regimen for locally advanced lung cancer and found that clinician corrections of autosegmentation generated by the Ethos ART platform improved the minimum dose to the planning target volume from 4.85 ± 3.03 Gy (*p* = 0.049) to 4.46 ± 8.99 Gy (*p* = 0.058) [35].

Overall, 78% of studies of the dosimetric effects of autocontouring have found either no statistical difference or a very small difference (clinically acceptable) in DVH metrics when comparing plans optimized with automated contours (deep learning or Atlas) with manual contours [28,29,30,31,32,33,36]. The other 22% of studies have shown a statistically significant dose difference between the dose obtained by using automated and manual contours [34,35]. However, the variations in conformality of the radiation dose either delivered to or in avoidance of the automated contoured structures make it difficult to evaluate the true impact of autocontouring across these studies. Additionally, all the studies investigated here used DVH parameters to assess the effects of autosegmentation on dosimetry. The subregions within each structure may also be important.

### 2.4. Efficiency Improvements from Autocontouring

Accelerating advancements in AI and machine learning autosegmentation tools have led to the need for evaluation metrics to assess their integration into routine clinical use [37,38,39]. Performance metrics have focused on the primary goals of autosegmentation, one of which is improving efficiency [38]. Efficiency can be defined as the relationship between healthcare resources and outcomes. For fixed costs, an increase in efficiency is weighed by maximizing outcomes. Optimally, healthcare outcomes are best assessed by final patient health output measures of overall survival, progression-free survival, and quality-adjusted life years. However, intermediate healthcare outputs, such as time spent per task, number of patients treated, and patient wait time, are also considered effective measures of efficiency outcomes [9,40,41]. Intermediate outputs of efficiency must appropriately balance both time and quality metrics [42,43]. Being in its infancy, the current focus of efficiency evaluation of autosegmentation has centered primarily on intermediate outputs mostly using metrics to assess time spent per task rather than final healthcare outputs. Studies reporting efficiency of autosegmentation of OARs and target volumes at different anatomic sites have shown improved efficiency outcomes when time per task metrics were used [42,44].

The overall concept of time efficiency is considered in two parts, one being direct reductions in primary segmentation and subsequent editing and the other ancillary time benefits resulting in more time for patient care, teaching, research, and tertiary healthcare metrics [9,40,42,45,46,47]. Much of the published literature on primary segmentation time reduction compares manual segmentation with Atlas-based or AI-based segmentation methods. Other reported methods have encompassed overall time from onset of commencing contouring (for manual segmentation) to a clinically acceptable final contour set, combining time taken for autosegmentation strategies and subsequent user-adjusted editing to an acceptable contour set (overall contouring time), or encompassing only ‘human-time’ involvement, which discounts initial autosegmentation time as clinicians can dedicate this to other aspects of healthcare. Autosegmentation-based solutions have been shown to reduce overall contouring time by 20% to 40% compared with manual segmentation [20,48,49,50].

### 2.5. Autocontouring and Predictions of Patient Outcomes

Various studies have assessed the effects of contour uncertainty on patient outcomes in terms of normal tissue complication probability (NTCP), tumor control probability (TCP), equivalent uniform dose (EUD), and overall survival. NTCP quantifies the risk of complications in an OAR with a specific biological endpoint when a given radiation dose is administered [51]. TCP is effectiveness of a certain radiation dose in controlling or eradicating a tumor [52]. EUD is defined as the amount of biologically equivalent dose that, when administered uniformly to a tumor, would induce the same cell kill as a non-uniform dose [53]. Finally, overall survival is the length of time that a patient remains alive after time of diagnosis or start of treatment. A high TCP combined with a low NTCP (i.e., a wider gap in the NTCP/TCP curve) is desired to maximize overall survival and quality of life.

Extensive studies of the impact of contour automation on TCP and NTCP are lacking. However, a few studies have attempted to correlate contour variations with outcome, which can shed light on how reduction in uncertainties through autosegmentation could affect NTCP and TCP. Jameson et al. investigated impact of IOV in delineating planning target volume (PTV) on TCP and EUD and found that geometric variation in PTV in the lateral (i.e., X or Z) direction was significantly related to TCP and EUD [54]. Moreover, deep-learning-based autosegmentation was shown to improve heart contouring, and dose metrics from automated contours showed stronger correlation with overall survival than their manual-contour-based metrics [34]. However, other studies have concluded that no significant correlation exists between contour variation and NTCP [55] or EUD [56]. Therefore, more work is needed to establish a relationship between automatic segmentation and patient outcomes, which relies on improved consistency in measurement of patient outcomes as well as consistency in segmentation.

## 3. Automated Treatment Planning

Treatment planning is the process of developing treatment machine parameters before treatment to deliver prescribed radiation to patients. Relevant machine parameters include radiation beam angles, beam weights, beam energy, couch angles, radiation field shaping, and more [57]. The goal of treatment planning is to accurately deliver prescribed radiation to the exact site of treatment to maximize tumor control with minimal damage to surrounding OARs. The tradeoffs between tumor control and safeguarding OARs are challenging and delicate to maintain because each patient presents with a unique set of anatomic geometries [57]. Therefore, treatment planning is often an iterative, time-consuming, and manual process.

Complexity of a manual treatment planning process can lead to significant variations in final treatment plans. Nelms et al. showed in an inter-institutional study that wide variability was present in treatment plan quality across treatment planners despite those planners being given very specific planning dose–volume histogram (DVH) objectives [58]. That study showed that variations in plan qualities could be attributed to planner skills rather than technological parameters or planner demographics [58]. Batumalai et al. showed that greater levels of planning experience translated to improvements in plan quality [59]. Variations in treatment planning are not without consequences. Moore et al. showed that suboptimal planning can lead to substantial excess risks of normal tissue complications [60]. Therefore, it is crucial but challenging to maintain consistently high-quality radiation treatment planning across the board when treatment planning processes are manual.

To reduce variations in manual treatment planning and to increase efficiency, investigators and vendors have introduced automatic treatment planning, including knowledge-based treatment planning (KBP) and non-knowledge-based treatment planning (non-KBP). KBP leverages planning information from prior patients to train mathematical models by using statistical inference. The models are then used to predict dose distributions for new patients [57,61]. KBP treatment planning techniques have been extensively researched, and many are commercially available (e.g., Eclipse’s RapidPlan) [57,62,63]. More recent advances in deep learning as a part of KBP enable faster and more accurate voxel-based dose predictions [57,64,65,66]. Non-KBP treatment planning uses heuristic rule implementation to mimic the manual planning process. Various studies have implemented non-KBP for treatment of cancers at different anatomic sites, including the whole brain, breast, cervix, and rectum [1,46,67,68,69,70]. Regardless of the method, automated treatment planning has been shown to improve consistency and efficiency of the planning process [69,71,72].

Clinical acceptability of treatment plans can be measured in several ways: by quantitative metrics, qualitative evaluations, and dosimetric effects. The following sections describe details of each of these methods of evaluation (quantitative, qualitative, dosimetric, efficiency gains, and predicted patient outcomes). 

### 3.1. Quantitative Evaluation of Autoplans

The DVH has been widely used for decades as the main means of quantitative evaluation of treatment planning [73]. DVH analysis assists in evaluating treatment plans by reducing a three-dimensional dose distribution to a one-dimensional graphic depiction of the dose distribution for various volumes of interest throughout the target volume and each OAR. DVH curves can be used to estimate target coverage and sparing of OARs, providing a clinically useful means of evaluating treatment plans [74].

In routine clinical practice, clinical goals are often quantified by DVH metrics. The most common DVH parameters used for evaluation include: (1) Dn%, the dose received by at least n% volume of a structure; (2) VdGy, the volume of a structure receiving at least d Gy of dose; (3) Dmax, the maximum dose; (4) Dmin, the minimum dose; (5) Dmean, the mean dose; and (6) Dmed, the median dose [75,76,77]. Nowadays, DVH metrics are used mainly for plan evaluation and optimization of routines (such as intensity-modulated radiation therapy (IMRT)) in most commercial treatment planning systems [78]. To achieve an optimal balance between target coverage and OAR sparing in IMRT or volumetric modulated arc therapy (VMAT), the final treatment plan is typically optimized by “tweaking” the DVH objectives until the plan is clinically acceptable [77,79].

Despite its obvious merits, use of the DVH for treatment plan evaluation has several limitations. The one-dimensional nature of the DVH translates into loss of spatial information on dose distribution within targets or OARs [80], meaning that different plans can generate nearly the same DVH for the same patient. Consequently, further evaluation of spatial distribution of dose relies heavily on manual visual inspection. Additionally, non-DVH metrics, such as anisotropic steepness of dose falloff, location, and magnitude of hot and cold spots, are also important and depend on the individualized treatment goals of the patient. Manual inspection has been found to be prone to IOV and can introduce personal biases [78,81,82]. Clinicians often determine the acceptability of a treatment plan based on its “look,” such as steepness of dose fall-off or location of hotspots, rather than its DVH metrics [83]. Moreover, selection of DVH parameters for plan evaluation and optimization relies heavily on personal judgment because of variability in prioritizing clinical goals in practice. Hence, no “gold standard” exists for DVH metrics that can indicate clinical acceptability of plans. Another limitation is that DVH accuracy depends on how the target and OARs are delineated [81]. Accuracy and consistency of clinical contours could lead to further uncertainties in DVH evaluation. Autocontouring may be one approach to introduce greater consistency in contours to help generate data to better inform future target DVH metrics.

Many other quantitative indices have been proposed to reduce subjectivity in plan evaluation based on DVH, including the conformity index, homogeneity index, EUD, and gradients index [78,84,85,86]. The conformity index was introduced by the RTOG to assess degree of congruence between a target and prescription isodose volume; on the other hand, the homogeneity index is used to evaluate non-uniform dose distribution inside targets [87]. The EUD was developed to generate a uniform dose for a target or an OAR that is biologically equivalent to a nonuniform dose distribution [86]. Recently, the gradient index, which accounts for the steep dose gradient outside the target volume, has been given high priority in stereotactic radiosurgery (SRS) and SBRT plans, in which heterogeneity is desirable [88]. These indices have been used together with DVH to support clinical assessments of treatment plan quality.

### 3.2. Qualitative Evaluation of Autoplans

The ideal in developing a radiation therapy plan is to generate an optimized dose distribution that fulfills two goals: complete coverage of targeted tumor volumes to the prescribed dose and the greatest possible sparing of normal tissues from irradiation. Radiation oncologists visually inspect generated treatment plans for their ability to deliver the prescribed radiation to target volumes. Conformity and coverage are key to ensuring that the affected tumor tissues are irradiated sufficiently and extensively to eliminate cancer, while care is taken to ensure that no area is too “cold” (under-irradiated) or “hot” (over-irradiated). Investigations of irradiation to be delivered to desired tissues must include ensuring that normal tissues adjacent to affected tumor volume are spared from irradiation. This inspection is an iterative process that often requires further optimization of plans to ensure that dose objectives are met to the greatest extent possible [89,90,91,92]. Known clinical and molecular-based features introduce further intricacies for evaluation of defined structures and irradiated volumes on the treatment-simulation images [93]. Thus, methods for ensuring structure and consistency in the review process are of growing importance for evaluation criteria for effective plan review.

#### 3.2.1. Dose Display and Calculation Options

Dose display and calculation options can substantially affect a physician’s decision-making process. The very first thing to consider is the computational grid upon which the dose is calculated. Although finer dose grids provide more accurate dose estimates, they require significantly more computational time and resources. The typical dose grid resolution varies from 1 to 3 mm [94,95] depending on site and type of treatment. The user needs to understand the effects of different dose grid resolution and choose them accordingly. The same dose distributions can lead to different decisions if the dose display options are different. Isodose and color wash are two widely used means of displaying dose distributions in two dimensions, and they each have their pros and cons. Isodose is better for displaying small hot and cold spots and for checking if OARs are abutting certain isodose lines, but selection of color table options (such as isodose lines to display) and the color scheme can distort information and mislead the user if the options were selected inappropriately. On the other hand, color wash is less dependent on color table options because it shows the dose variation as a color spectrum but may not clearly indicate hot and cold spots. Reviewers should understand the properties of each display option and need to use the same options consistently during the review process.

#### 3.2.2. Graphical Methods

To expedite the process of plan review, visual methods have been proposed to represent relevant dosimetric information and desired clinical objectives. Commercial treatment planning systems have incorporated side-by-side plan comparison viewing panes of DVH and dose distributions. Although these representations enable historical comparisons or potential coverage improvements between plans, they are limited in both scope and depth of plan information available for comparison. Ventura et al. investigated a novel approach of integrating dose constraints with weighted scoring of physician preferences in a graphical radar plot representation, enabling plans to be ranked according to prescribed dose constraints and clinical validation criteria [90,96]. Additional visualization approaches have leveraged the power of retrospective DVH data to provide visual histogram analysis of clinical plans to compare generated plans with manually generated plans [97,98].

#### 3.2.3. Evaluation Rubrics

Before radiation therapy plans are accepted for patient treatment, model outputs must first be validated for consistency and adherence to clinical practice. Scorecards and checklists are structured performance tools used to gauge a plan’s adherence to specific dose–volume objectives and constraints for evaluating plan acceptability [89]. In one study, checklists were found to improve the rate of error detection in plans, with a reported increase in detection rate of 20% [99]. Autoplans are commonly reviewed by at least three radiation oncologists, either from the same institution or across different institutions, with overlapping fields of expertise in treatment of the particular disease site. The reviews are scored on a three- to five-point rating scale that deems the plan “Acceptable”, “Unacceptable”, or in need of minor/major revisions [96,100]. The number of ratings used to assess plans can vary depending on treatment site or on level of response scaling desired for reporting physician reviews. These rating systems provide a uniform grading scale on which physicians can comment on degree of plan acceptability for converting planning nuances and expertise into comparable categorical information.

#### 3.2.4. Pitfalls in Subjective Evaluation

Review of radiation therapy plans depends on the expertise of the reviewer. Quality assurance (QA) measures, such as ‘chart rounds’ with practicing peers, are becoming common practice to increase both the skills of the reviewers and the standard of the delivered radiation therapy. A longitudinal study spanning approximately 5 years demonstrated the benefits of peer-review systems on improving plan quality and reducing the need for significant plan modifications before treatment [101]. However, not all needed plan modifications are detected via these review processes. A study of error detection rates in institutional peer review during physician chart rounds revealed that 45% of simulated treatment plan errors were undetected. Time taken to review plans and the point at which they were reviewed (either earlier or later in the review process) were thought to affect error detection rates, suggesting that there is still room for improvement in the plan review process [102].

Although the manual review process is the “gold standard” for evaluating radiotherapy plans, the review process is always subjective and can differ considerably from physician to physician. This inter-physician variability is the biggest hurdle for determining clinically acceptable plans, and, as a result, multiple physicians review the same plans in many of the plan evaluation studies [70,100]. Gronberg et al. [100] asked three physicians to select the preferred plan for 20 pairs of head-and-neck radiotherapy plans and all three physicians selected the same plan only half of the time. Even assessment by the same physician of the same plan can change over time. According to Ventura et al. [96], intra-physician variability in plan selection was actually higher than inter-physician variability for nasopharynx cases. These studies indicate that the review process can be influenced by a variety of factors (as noted in this chapter) that can, in turn, introduce inter- and intra-variability in physician decisions [96].

### 3.3. Efficiency Improvements from Autoplanning

Quantitative assessment of time savings from autoplanning is often considered in terms of actual human resource hours saved on an autoplan versus a manual plan [103]. The time saved depends on the complexity of the plan that is being generated (3D conformal radiation therapy, IMRT, or VMAT). Complexity of disease site also affects how quickly the autoplan is generated and reviewed; for example, head and neck plans are complex and often require numerous targets and corresponding dose levels. Several studies have shown that manual planners required from 33 min to 2.5 h to generate a plan, whereas autoplans can be generated in as little as 20 s for a deep learning algorithm to 15 min for KBP [104,105]. Although different studies take different approaches to quantifying amount of time saved with autoplanning, the important point to note is that automated planning often does not require user intervention. This means that a user can tend to other necessary tasks while waiting for the plan to be generated. Different approaches to quantify efficiency of autoplanning are outlined in the following paragraphs.

Kaderka et al. used KBP to produce treatment plans for the prostate and prostatic fossa, head and neck, and hypofractionated lung disease [106]. To clarify the effect of KBP on overall workflow efficiency, these investigators compared manual planning time with KBP + human planning time across each disease site. Statistical differences between manual and autoplanning time were quantified with two-tailed Mann–Whitney U tests. This study revealed that KBP significantly reduced the planning time for prostate, prostatic fossa, and lung disease sites. Further comparisons of the dose achieved before and after user intervention revealed no significant differences in dose to OARs in the prostate, prostatic fossa, and lung disease sites, but adding human intervention to refine the plans for head-and-neck cancer led to clinically relevant dose reductions. These findings led these authors to refine their KBP model to improve the dosimetric results.

Combining autocontouring with autoplanning has the potential to maximize efficiency in terms of manpower hours; however, it is difficult to directly compare manual planning time with automated planning time. Information on time needed for end-to-end solutions has been reported. In one such study, Rhee et al. combined deep-learning-based autocontouring with autoplanning algorithms to achieve 90% clinical acceptability across three different planning techniques for cervical cancer [70]. The average time (±1σ) to create a plan was 9.2 ± 2.3 min for four-field box plans, 8.5 ± 2.6 min for 3D conformal radiation therapy plans, and 46.6 ± 8.6 min for VMAT plans. Optimization was included in the reported planning time. Hernandez et al. contoured 16 normal tissue structures on full-body scans for pediatric craniospinal irradiation in an average of 20 min, and the resulting treatment plan with optimization was generated in an average of 3.50 ± 0.4 min [67]. All studies emphasized that the reported time was “without user intervention” time, meaning that the user could tend to other tasks while waiting for the automated algorithms to run.

Each of these studies assessed clinical acceptability of autoplans by using a 5-point Likert scale to add qualitative metrics to plan quality. The autoplanning time reported in all three studies did not include time required for necessary clinical edits. Although classification of minor or major edits is a subjective process, all studies provided examples of each kind of edit. For example, a minor edit could be changing the position of a multileaf collimator to increase or decrease coverage. Providing examples of autoplan edits and clear instructions of what changes would be made to the plan before its use is an excellent way to provide context for time needed for required edits. In most cases, editing an autoplan is a more efficient planning approach than creating a new plan from scratch.

### 3.4. Autoplanning and TCP/NTCP Evaluation

Perhaps the most meaningful way to evaluate radiation therapy plans is by predicted patient outcomes. As noted previously, optimal plans deliver a tumoricidal dose while minimizing adverse side effects. Calculations of TCP and NTCP, based on TCP and NTCP models, can help to guide plan optimization and assessment. The many existing TCP and NTCP models can generally be categorized as mechanistic or empirical. Most TCP models combine Poisson statistics with a linear–quadratic dose–response model and are derived from the assumption that all tumor cells must be killed to achieve tumor control [107,108]. NTCP models attempt to relate complex dosimetric information with probability of occurrence of an acute or late side effect. The Quantitative Analysis of Normal Tissue Effects in the Clinic (QUANTEC) reports published in 2010 summarized published NTCP models for 16 organs [109].

Despite the clear advantages of using TCP and NTCP modeling to optimize and assess plans, uptake in the radiation oncology community has been slow. A report from an American Association of Physicists in Medicine (TG-166) warns of the potential dangers of implementing biologically related models into treatment planning and the importance of understanding the limitations of the models. One major limitation of most models is their being based on DVH parameters [109]. As noted previously, DVHs do not capture complex 3D dose information or tissue heterogeneity. Moreover, they are calculated from a planning simulation CT scan and do not account for variation in planned versus delivered dose distribution. Another limitation arises from use of simplistic models to describe complex patient outcomes. Many models have only dosimetric inputs and do not consider clinically relevant patient characteristics. On the other hand, models that include many variables may result in overfitting and reduced generalizability. In response to the challenges faced in interpreting and aggregating the current literature, QUANTEC released recommendations for study methodology and reporting for normal tissue complications [110].

Despite the limitations in outcome modeling, the need remains for assessing plan quality through predicted and reported patient outcomes. Although automated treatment planning can be assessed via quantitative plan metrics (e.g., dose–volume metrics) that are believed to correlate with patient outcomes, predicting or measuring the outcome itself would more closely capture the effects on patients’ quality of life. Moore et al. demonstrated the potentially large benefits of using KBP to improve the quality of clinical trial plans [60]. They validated a Lyman–Kutcher–Burman model, the most widely used and well known NTCP model in the United States, to predict grade ≥2 rectal complications in clinical trial RTOG 0126. They then compared their predicted rectal DVHs with those of the clinical trial plans to retrospectively estimate excess risk of normal tissue complications. This retrospective analysis showed that about 43% of patients treated in that trial were at excess risk of >5% for grade 2+ late rectal toxicities. Fried et al. compared treatment plans for oropharyngeal cancer guided by predicted DVHs in a prospective analysis of baseline (before implementation of the DVH prediction tool) versus DVH-guided clinical trial plans at their institution [111]. They demonstrated advantages of DVH-guided planning that used both dosimetric endpoints and patient-reported outcomes. This is one of the first tests of automated planning approaches that incorporate patient outcomes, and hopefully it will serve as an example for future studies.

## 4. Other Considerations

The focus of the previous sections has been on evaluating autocontours and autoplans in terms of quality (clinical acceptability) of output. The following section considers some other aspects of ‘clinical acceptability’ that are important for introducing a new tool into clinical use, including automation bias, interface design, off-label use, risk assessment, and ethics.

### 4.1. Automation Bias

Risk is important to consider when evaluating clinical acceptability. One type of risk is overreliance on automation, otherwise known as automation bias. This occurs when clinicians develop a high level of trust for artificial intelligence (AI), which introduces bias in favor of advice from an automated system over their own decisions or advice from their peers. This bias can lead clinicians to reverse their own (correct) decisions in favor of (incorrect) advice provided by an automated system [112]. One study of how use of a computerized diagnostic system affected clinical decision-making showed that, in 6% of cases, clinicians failed to act on their own correct decisions, choosing instead to follow the erroneous advice from the computerized diagnostic system [113]. Possible methods to prevent automation bias include making users aware of the AI reasoning process [114], emphasizing human accountability [115], presenting uncertainties in AI output to users [116], and training users on situation-specific reliability [117].

### 4.2. Benchmark Datasets and Interface Design

The first and most important concern in commissioning autosegmentation tools for clinical use is segmentation accuracy. Benchmark datasets are needed to assess segmentation accuracy. Benchmark datasets can be prepared from local institutions; however, such datasets usually require excessive time to curate data for numerous anatomical sites [118], leading to a trend toward community-wide testing with common datasets [119]. A first step in the process of providing limited-size datasets with clearly defined metrics for evaluation is being addressed through the “Grand Challenge” program, a platform for end-to-end development of machine learning solutions for biomedical imaging [120,121,122,123]. Public data repositories, such as the National Cancer Institute’s Cancer Imaging Archive [124], provide well curated data for benchmarking; however, variations in inter-institutional data in such archives could influence the analytical results. Thus, evaluation metrics are needed to quantify accuracy of segmentation, and their meaning and details of their implementation must be well understood. Individual quantitative evaluation metrics may not directly relate to human perception; rather, a combination of different evaluation metrics should be considered for the evaluation, and the evaluation should account for inter-observer variability if possible [125]. In one recent study, differences in implementation of evaluation metrics resulted in large discrepancies in reported results, and many in-house implementations were found to have “bugs” in some measures [126]. In addition to segmentation accuracy, workflow integration or interface design should be considered in clinical commissioning, and this is often an important limiting factor for using autosegmentation tools [125]. Workflow integration should consider clinical applications, e.g., treatment planning or adaptive planning. Another aspect to consider is the need to integrate the autosegmentation tool with existing software, with easy access from the treatment planning system and easy retrieval of segmentation results. Another factor is flexibility in use of the tool. In clinical use, “one-button clicking” is usually preferable. For research purposes, some flexibility should be offered in terms of ability to tune the algorithm parameters, among other considerations.

### 4.3. Off-Label Use

In the United States, approval of medical devices for specific medical uses is completed by the US Food and Drug Administration (FDA). “Off-label” use of a drug or other medical device refers to its being administered in a way that has not been approved by this agency [127]. Using any medical device in ways not approved by the FDA that lead to harming patients can render the provider legally responsible [128]. Tort law deals with civil wrongs committed by one party that causes harm to another party. The legal liability of healthcare providers for harms caused by standard-of-care and off-label use of AI has been suggested to be similar to that for other approved medical devices [128]. Physicians who receive non-standard-of-care recommendations from AI may face greater legal liability than physicians who do not receive AI recommendations, and this issue requires a great deal of further study [129]. As use of AI continues to emerge in a wide variety of medical fields, more guidance and policies from legal bodies are needed for healthcare providers to understand associated risks [130].

### 4.4. Failure Modes and Effects Analysis

To effectively minimize risk when introducing automated tools into clinical practice, several groups have used a prospective method of risk assessment called failure modes and effect analysis (FMEA). Wexler et al. used FMEA to compare two methods for commissioning a treatment planning system: one manual and one using an automated commissioning process [131]. Through this study, they were able to confirm that the automated method, which used application programming interface (API) scripting, preloaded beam data, and digital phantoms, increased the safety of commissioning these systems.

Kisling et al. used FMEA to evaluate the procedures for deploying an automated contouring and treatment planning tool [132]. Risk was evaluated in two different scenarios: first, with plan QA addressed according to standard manual processes, and, second, with an automated QA program integrated into the system. The results showed that integrating the automated QA tools into the planning workflow led to decreases in both the maximum and mean risk scores, indicating that automated plan QA could effectively increase patient safety.

Nealon et al. also showed that FMEA could be used to identify and subsequently limit risks associated with using automated planning tools [133]. In that analysis, errors were identified and participants then worked to reduce the associated risks by updating the software interface, the QA process, and the user training materials. Use of the FMEA process during development of this automated planning tool ultimately resulted in the final software being safer, with fewer occurrences of human and software failures.

### 4.5. Ethics

Although treatment planning algorithms may seem agnostic to ethical biases, algorithms can perpetuate biases that are inherent to a set of training data. Several considerations in algorithm development, if overlooked, can exacerbate inequities between patient populations. Chen et al., in their review of the ethical concerns associated with use of machine learning in healthcare, listed several examples that we consider to be directly relevant to algorithm development and deployment in radiation oncology: (1) using confounding features that cause incorrect associations, (2) naïve inclusion of race as a machine learning feature, (3) overfitting when the data lack diversity, (4) deployment of the algorithm for a patient population that differs from the training population, and (5) model auditing or QA [134]. In addition to these, there are clinical practice variations that introduce biases in models that can affect ‘clinical acceptability’. Because ethical biases in treatment planning have not been thoroughly studied, we strongly recommend that developers and researchers pursue diligence in such areas so that treatment planning algorithms operate robustly and without bias. One way to directly apply this principle is by being transparent regarding makeup of patient populations in training and benchmark datasets. Transparency is especially important for datasets that are to be made public so that the patient population details are sufficient to enable algorithms that are competing for state-of-the-art or benchmarking performance to be appropriately stewarded for safe and accurate use of the data by the global clinical and scientific community.

## 5. Conclusions and Recommendations

The concept of clinical acceptability is vaguely defined and lacks standardized metrics for its assessment. In this review, we have described the many approaches that can be used to try to understand clinical acceptability of new autocontouring and autoplanning tools.

The most common approach to assess quality of autocontouring tools is to use geometric overlap metrics (Dice similarity coefficient, etc.), comparing the output of the tool with manually drawn “expert’ contours. The advantage of this approach is its accessibility as it can be performed by any user with sufficient data. The main disadvantage is that these metrics are generally poorly correlated with arguably more important factors, such as physician review or impact of differences on the patient. A higher value of DSC, for example, does not necessarily indicate a “better” contour. In fact, studies have shown that “excellent” DSC values (>0.90) can correspond to multiobserver agreement that the contour is not clinically acceptable [25]. These comparisons are further complicated as the original clinical contouring can be influenced by factors beyond the primary images, including additional imaging results (e.g., from PET images) and the results of physical exam or endoscopic examination. These limitations should be considered when making conclusions on the quality of an autocontouring tool. For these reasons, whenever possible, we suggest that these quantitative metrics should be accompanied by physician review as this may highlight errors in contours that may be hidden in, for example, box plots of DSC values. Both these approaches are limited by inter-observer variations in how contours are drawn, and this issue should be considered when drawing conclusions from experimental results. Studies have also demonstrated the potential role of internal bias against AI in clinician assessment of AI-driven results [135]. After deployment, automation bias, where users are overreliant on automated contours, can also be a concern.

Assessment of autocontours should also consider how they will be used and with which patient population. The training and test patient populations should be well described (tumor staging, etc.). Testing should then consider application of autocontours. For example, if the autocontours are being used as part of an autoplanning process, we suggest going one step further and determining the impact of the autocontours on the treatment plan: for example, by creating a treatment plan based on the autocontours and assessing the doses delivered to the true contours, which may be the clinical contours or, even better, contours that have been reviewed and approved by a group of experts. That is, the actual use-case of the autocontours plays an important part in the assessment. This does not, however, negate use of simpler assessment approaches (DSC, etc.) as they may highlight different quality issues that could become important if the contours are used for a different application (i.e., not as part of autoplanning).

Autoplanning is generally assessed in similar ways. Quantitative assessment of plan quality, typically using DVH-metrics, is reasonably closely linked to “clinical acceptability”, so it is a reasonable approach. Although not as limited as geometric metrics for autocontours, the DVH metrics do not tell the whole story about “clinical acceptability”, and we recommend that these quantitative metrics be accompanied by physician review. As with autocontours, this may highlight errors that are not clear in the quantitative assessment—for example, dose spillage into unexpected or unwanted parts of the body. It is particularly important to consider the number of reviewers as a single reviewer’s opinion is not likely to be representative of all physicians. Understanding the correlation and translational impact of errors between geometric metrics, dosimetric metrics, clinical decisions, and outcomes is an important area of research that needs to be investigated. While unnecessarily overdosing normal tissue or underdosing the tumor should never be knowingly accepted, small variations between autocontours and manual contours will continue to exist as there exist variations between manual contours drawn by experts and between their own contours! These small variations should not restrict the potential significant benefits to autocontouring and autoplanning.

Not all developers or users have the resources to follow all these suggestions; physician review, in particular, takes a substantial amount of time, which can be difficult to achieve. In the absence of more advanced assessment approaches, we suggest that a developer/user acknowledge and discuss these limitations in any publications or reports. These limitations, and the need to overcome them, also highlight the importance of data sharing, “challenges” run by professional and research societies, and national databases, such The Cancer Imaging Archive, to enable all developers to systematically evaluate their results.

It is also important to recognize the role of clinical information not present in images (e.g., the results of physical examination) that may influence contouring and planning decisions of a clinical team. It is important to review the discrepancies between the automated tools and the clinical results in the full clinical context to determine what potential role these additional data may play in achieving the optimal clinical results.

Depending on the application and goal of the tool, it may be appropriate to consider other factors. For example, software development and deployment should always include risk assessment and usability studies. Timing studies may also be useful in determining overall clinical acceptability of new tools.

## Figures and Tables

**Table 1:** Five-point Likert scale used to validate an autosegmentation model deployed at MD Anderson Cancer Center.

Likert Scale		Explanation
5	Strongly agree	Use-as-is (i.e., clinically acceptable, and could be used for treatment without change)
4	Agree	Minor edits that are not necessary. Stylistic differences, but not clinically important. The current contours/plan are acceptable.
3	Neither agree or disagree	Minor edits that are necessary. Minor edits are those that the reviewer judges can be made in less time than starting from scratch or are expected to have minimal effect on treatment outcome.
2	Disagree	Major edits. This category indicates that the necessary edits are required to ensure appropriate treatment, and sufficient significant that the user would prefer to start from scratch.
1	Strongly disagree	Unusable. This category indicates that the quality of the automatically generated contour or plan are so bad that they are unusable.

## Data Availability

Not applicable.

## Abbreviations

3DCRT	Three-dimensional conformal radiation therapy
ART	adaptive radiation therapy
Dmean	Mean dose to a structure
Dmed	Median dose to a structure
DSC	Dice similarity coefficient
DVH	Dose–volume histogram
EUD	equivalent uniform dose
FMEA	failure modes and effect analysis
GAN	general adversarial network
Gy	Gray (unit of radiation dose)
HD	Hausdorff distance
IMRT	Intensity-modulated radiation therapy
IOV	interobserver variability
KBP	knowledge-based treatment planning
MSD	mean surface distance
NTCP	normal tissue complication probability
OAR	organs at risk
QA	Quality assurance
QUANTEC	Quantitative Analysis of Normal Tissue Effects in the Clinic
RTOG	Radiation Therapy Oncology Group
SBRT	stereotactic body radiosurgery
SRS	stereotactic radiosurgery
TCP	tumor control probability
VMAT	Volume-modulated radiation therapy
